# Variety and quantity of dietary insoluble fiber intake from different sources and risk of new-onset hypertension

**DOI:** 10.1186/s12916-023-02752-7

**Published:** 2023-02-16

**Authors:** Ziliang Ye, Qimeng Wu, Sisi Yang, Yanjun Zhang, Chun Zhou, Mengyi Liu, Zhuxian Zhang, Panpan He, Yuanyuan Zhang, Rui Li, Huan Li, Chengzhang Liu, Jing Nie, Fan Fan Hou, Xianhui Qin

**Affiliations:** 1 Division of Nephrology, National Clinical Research Center for Kidney Disease, State Key Laboratory of Organ Failure Research, Guangdong Provincial Institute of Nephrology, Guangdong Provincial Key Laboratory of Renal Failure Research, Nanfang Hospital, Southern Medical University, Guangzhou, 510515 China; 2 Department of Epidemiology and Biostatistics, School of Public Health, Anhui Medical University, Hefei, 230032 China; 3 Institute of Biomedicine, Anhui Medical University, Hefei, 230032 China

**Keywords:** Dietary insoluble fiber intake, Variety, Quantity, Different food sources, New-onset hypertension

## Abstract

**Background:**

The relations of the variety and quantity of different sources of dietary insoluble fibers and hypertension remain uncertain. We aimed to investigate the associations between the variety and quantity of insoluble fibers intake from six major food sources and new-onset hypertension, using data from the China Health and Nutrition Survey (CHNS).

**Methods:**

Twelve thousand one hundred thirty-one participants without hypertension at baseline from CHNS were included. Dietary intake was measured by three consecutive 24-h dietary recalls combined with a household food inventory. The variety score of insoluble fiber sources was defined as the number of insoluble fiber sources consumed at the appropriate level, accounting for both types and quantities of insoluble fibers. The study outcome was new-onset hypertension, defined as blood pressure ≥ 140/90 mmHg, or physician-diagnosed hypertension or receiving antihypertensive treatments during the follow-up.

**Results:**

During a median follow-up of 6.1 years, 4252 participants developed hypertension. There were L-shaped associations of dietary insoluble fibers derived from vegetables, beans, tubers, and fruits with new-onset hypertension; a reversed J-shaped association of whole grain-derived insoluble fiber with new-onset hypertension; and no obvious association of refined grain-derived insoluble fiber with new-onset hypertension. Therefore, refined grain was not included in the insoluble fiber variety score calculation. More importantly, a higher insoluble fiber variety score was significantly associated with lower risks of new-onset hypertension (per score increment, hazard ratio, 0.50; 95% CI, 0.45–0.55).

**Conclusions:**

There was an inverse association between the variety of insoluble fibers with appropriate quantity from different food sources and new-onset hypertension.

**Supplementary Information:**

The online version contains supplementary material available at 10.1186/s12916-023-02752-7.

## Background

Hypertension is a major cause of the cardiovascular disease (CVD) and all-cause mortality worldwide [1]. The prevalence of hypertension is still rising rapidly in low and middle-income countries [2]. An estimated 626 million women and 652 million men aged 30–79 years were living with hypertension globally in 2019 [3]. Therefore, it is of great clinical significance to identify more modifiable factors for the primary prevention of hypertension.

Recently, there has been growing interest in studies of nutrients and the risk of hypertension [4, 5]. Dietary fiber, composed of indigestible and non-absorbable carbohydrate polymers, is found primarily in plant material in the diet. Previous studies have reported that the amount and composition of dietary fibers differ from food to food [6]. Consistently, studies have shown that the beneficial effect of dietary fibers on CVD varied significantly with its water solubility and food sources [7, 8]. However, although a previous meta-analysis of randomized control trials (RCTs) found that higher consumption of beta-glucan fiber (soluble fiber) was associated with lower blood pressure (BP) levels [9], none of the previous studies have examined the relation of total dietary insoluble fiber, or insoluble fibers from specific food sources, with the risk of new-onset hypertension. Furthermore, a previous cross-sectional study showed that dietary fiber from different food sources had different associations with the prevalence of hypertension [10]. Nevertheless, to date, the relation of the variety of insoluble fiber sources with new-onset hypertension has not yet been examined.

To address these essential knowledge gaps, the current study aimed to evaluate the prospective associations between the variety and quantity of insoluble fibers intake from six major food sources (whole and refined grains, vegetables, beans, tubers, and fruits) and new-onset hypertension in general Chinese adults., using data from the China Health and Nutrition Survey (CHNS), a national health and nutrition survey in China.

## Methods

### Study design and participants

The study design and major results of the CHNS have been reported elsewhere [11–15]. In brief, CHNS is an ongoing nationwide multipurpose longitudinal open cohort study established in 1989 and has been followed up every 2 to 4 years with a sum of 10 rounds already completed (1989, 1991, 1993, 1997, 2000, 2004, 2006, 2009, 2011, and 2015). The study participants were sampled from 9 provinces (Heilongjiang [enrolled in 1997], Liaoning, Shandong, Henan, Jiangsu, Hubei, Hunan, Guizhou, and Guangxi) and 3 of China’s largest autonomous cities (Beijing, Shanghai, and Chongqing [all enrolled in 2011]) with a multistage, random cluster approach. In the 2009 round of CHNS, blood samples were collected. All samples were analyzed with strict quality control at a national central lab in Beijing (medical laboratory accreditation certificate ISO 15189:2007). By 2011, the survey included 12 provinces/autonomous cities and 288 communities, which covered 47% of China’s population [11].

A prospective open cohort study design was employed in our current study based on 7 rounds of CHNS data from 1997 to 2015. As shown in Additional file 1: Figure S1, we first excluded participants who were pregnant (360 person-waves) or < 18 years old (17,672 person-waves). Among the remaining 76,500 person-waves, the participants with BP data measurements (69,852 person-waves) did not differ in most of the baseline characteristics from those with missing BP data (6648 person-waves; Additional file 1: Table S1). The remaining participants who were surveyed at least two rounds were included, and the first round is termed as the baseline. In addition, participants who had hypertension (defined as having systolic blood pressure [SBP] ≥ 140 mm Hg and/or diastolic blood pressure [DBP] ≥ 90 mm Hg, previously diagnosed by physicians, or currently receiving antihypertensive therapy) and self-reported physician-diagnosed cardiovascular diseases at baseline, missing cumulative average dietary insoluble fiber data, or implausible cumulative average dietary energy data (male: > 4200 or < 600 kcal/day; female, > 3600 or < 500 kcal/day) [16] were also excluded. Finally, a total of 12,131 participants were included in the final analyses (Additional file 1: Figure S1).

Data and study materials that support the findings of this study can be found on the CHNS official website (http://www.cpc.unc.edu/projects/china). The study was approved by the institutional review boards of the University of North Carolina at Chapel Hill and the National Institute of Nutrition and Food Safety and Chinese Center for Disease Control and Prevention. Each participant provided written informed consent.

### Dietary nutrient intakes

In each survey round of CHNS, trained nutritionists collected dietary data through face-to-face interviews. Individual diet assessment was repeatedly assessed with 3 consecutive 24-h dietary recalls at an individual level in combination with weighing inventory over the same 3 days at the household level. The 3 consecutive days were randomly allocated from Monday to Sunday and were almost equally balanced across the 7 days of the week for each sampling unit. The China food composition tables (FCTs) were used to calculate nutrient intakes of each participant. It has been validated that 24-h dietary recall could accurately assess energy and nutrient intake [17–19]. The amount of dietary insoluble fiber for each food, which was measured with the neutral detergent method, was available from the Chinese FCTs.

We calculated 3-day average intakes of dietary macro- and micro-nutrients in each round in the analyses. To represent long-term dietary intake and minimize within-person variation, all values of each nutrient in the analyses, if not specified, were presented as the cumulative averages, using all results from baseline to the last visit before the date of new-onset hypertension, or using all results during the follow-up among participants without new-onset hypertension. Moreover, in our current study, total insoluble fiber was divided into specific sourced fibers. Food sources constituting these subtypes are presented in Additional file 1: Table S2. The variety score of insoluble fiber sources was the sum of the total numbers of the major food sources of insoluble fibers consumed at the appropriate quantity during the study period [20, 21]. The appropriate quantity for each major food source of insoluble fiber was determined by assessing insoluble fiber intakes from different food sources as categorical variables (quartiles or tertiles) and choosing the corresponding insoluble fiber categories with the relatively lowest risk of new-onset hypertension.

### Assessment of blood pressure and covariates

Seated BP was measured by trained research staff using a mercury manometer and an appropriate-sized cuff, following a standard method at each study survey after the participants had rested for 5 min. The BP of the same arm was measured three times in a quiet and bright room. The mean SBP and DBP of all measurements were used in the analysis.

Demographic and lifestyle information was available through questionnaires, including age, sex, smoking status, occupations, education levels, and living regions. Calibrated equipment was used to measure body height and weight following a standard operation procedure. Body mass index (BMI) was calculated as weight (kg) divided by height squared (m^2^).

### Assessment of outcomes

The study outcome was new-onset hypertension, defined as an SBP ≥ 140 mmHg or a DBP ≥ 90 mmHg, or physician-diagnosed hypertension, or receiving antihypertensive treatments during the follow-up [22, 23].

The year of each participant's first entry into the survey was considered as the baseline. The follow-up person-time for each participant was calculated from the baseline date until the first hypertension diagnosis (the middle date between the survey of the first diagnosis and the nearest survey before), the last survey round before the participant’s departure from the survey, or the end of the latest survey (2015), whichever came first.

### Statistical analysis

Population characteristics were presented as mean (standardized deviation [SD]) or proportions for continuous and categorical variables, respectively. Differences in population characteristics by the quartile of dietary total insoluble fiber intake were compared using ANONA tests or ${\chi }^{2}$ tests, accordingly.

Age-stratified Cox proportional-hazards models were used to estimate the association of variety score of insoluble fiber sources, intake of total insoluble fiber, and insoluble fibers from different food sources (refined and whole grains, vegetables, beans, tubers, and fruits) with new-onset hypertension, without and with adjustments for sex, BMI, SBP, DBP, smoking, alcohol drinking, urban or rural residents, regions, education levels, occupations, physical activity levels, self-reported physician-diagnosed diabetes, dietary intakes of sodium, potassium, protein, fat, and carbohydrate. Moreover, mutual adjustments for intakes of other sources of insoluble fiber were further included for the association between insoluble fibers from different food sources and new-onset hypertension. The Schoenfeld residual test was used to test the proportional hazards assumption, and no clear evidence of violation was detected. We also used restricted cubic splines with 3 knots (25%, 50%, 75% of intake of insoluble fibers) to investigate the potential nonlinear relationship of total dietary insoluble fiber and insoluble fibers from different food sources with new-onset hypertension with the adjustments for the covariates mentioned before.

A series of sensitivity analyses were conducted. First, dietary intakes of vitamin A, vitamin B_2_, niacin, copper, and zinc were further adjusted. Second, dietary intakes of refined and whole grains, vegetables, beans, tubers, and fruits were further adjusted. Third, BMI trajectory classes, which were estimated by the R package lcmm, were further adjusted. Fourth, total insoluble fiber intake was estimated by multiple source methods (MSM) [24]. Fifth, those with physician-diagnosed hypertension or receiving antihypertensive treatments were further excluded to account for the effect of possible dietary changes due to diagnosis on the results. Moreover, possible modifications of the association between variety score of insoluble fiber source and new-onset hypertension were evaluated by stratified analyses and interaction testing.

A 2-side *P* value < 0.05 was considered statistically significant. All statistical analyses were conducted using R software (version 4.0.2, https://www.R-project.org/).

## Results

### Study participants and baseline characteristics

Of the included 12,131 participants (Additional file 1: Figure S1), the mean (SD) of dietary total insoluble fiber intake was 10.8 (6.4) g/day. Refined grains followed by vegetables, beans, whole grains, tuber, and fruit are the major sources of insoluble fiber, constituting more than 90% of dietary insoluble fiber intake (Additional file 1: Table S2). The mean (SD) age was 41.1 (14.1) years, and 6459 (53.2%) of the participants were female.

The baseline characteristics of study participants were presented according to dietary insoluble fiber intake quartiles in Table 1. Participants with higher dietary insoluble fiber intake were more likely to be male, physically active, smokers, alcohol drinkers, and less likely to be urban residents, unemployed; had lower education levels, higher intakes of protein, fat, carbohydrate, sodium, and potassium; and had lower intake ratio of sodium-to-potassium.

**Table 1:** Participants characteristics by quartiles of total dietary insoluble fiber intake

Variables	Quartiles of dietary insoluble fiber, g/day				*P* value
	**Q1(< 7.1)**	**Q2(7.1–9.6)**	**Q3(9.6–12.9)**	**Q4(> 12.9)**	
***N***	3033	3032	3033	3033	
Male	1204 (39.7)	1371 (45.2)	1422 (46.9)	1675 (55.2)	< 0.001
Age, years	43.5 (15.8)	40.8 (13.6)	39.8 (13.2)	40.4 (13.6)	< 0.001
Body mass index, kg/m^2^	22.4 (3.1)	22.3 (3.1)	22.4 (3.1)	22.5 (3.0)	0.160
Systolic blood pressure, mmHg	114.5 (11.8)	113.7 (11.4)	113.1 (11.4)	114.3 (11.1)	< 0.001
Diastolic blood pressure, mmHg	74.2 (7.7)	74.0 (7.9)	74.2 (8.0)	74.4 (7.8)	0.161
Follow-up duration, years	6.0 (4.5)	8.2 (5.4)	8.8 (5.4)	8.2 (5.4)	< 0.001
Smoking, *n* (%)	814 (26.9)	882 (29.3)	896 (29.7)	1076 (35.7)	< 0.001
Alcohol drinking, *n* (%)	872 (29.0)	1001 (33.4)	1035 (34.6)	1219 (40.8)	< 0.001
Urban resident, *n* (%)	1368 (45.1)	1170 (38.6)	988 (32.6)	853 (28.1)	< 0.001
Self-report diabetes, *n* (%)	62 (2.1)	36 (1.2)	25 (0.8)	27 (0.9)	< 0.001
**Regions, *n* (%)**					< 0.001
Central	1220 (40.2)	1184 (39.1)	1417 (46.7)	1742 (57.4)	
North	462 (15.2)	609 (20.1)	794 (26.2)	604 (19.9)	
South	1351 (44.5)	1239 (40.9)	822 (27.1)	687 (22.7)	
**Occupation, *n* (%)**					< 0.001
Farmer	627 (20.8)	950 (31.7)	1298 (43.4)	1466 (48.8)	
Worker	363 (12.1)	412 (13.7)	347 (11.6)	335 (11.2)	
Unemployed	1052 (34.9)	731 (24.4)	635 (21.2)	589 (19.6)	
Others	970 (32.2)	904 (30.2)	710 (23.7)	612 (20.4)	
**Education, *n* (%)**					< 0.001
Illiteracy	534 (17.9)	465 (15.6)	551 (18.6)	641 (21.6)	
Primary school	507 (17.0)	580 (19.5)	626 (21.1)	604 (20.4)	
Middle school	970 (32.5)	990 (33.2)	998 (33.6)	1021 (34.4)	
High school or above	978 (32.7)	943 (31.7)	792 (26.7)	701 (23.6)	
**Physical activity, *n* (%)**					< 0.001
Low	1189 (39.5)	979 (32.6)	891 (29.7)	948 (31.6)	
Moderate	1054 (35.0)	1071 (35.7)	974 (32.4)	908 (30.2)	
Vigorous	768 (25.5)	951 (31.7)	1140 (37.9)	1148 (38.2)	
**Dietary intake**					
Insoluble fiber, g/day	5.4 (1.3)	8.3 (0.7)	11.1 (1.0)	18.4 (8.2)	< 0.001
Energy, kcal/day	1826.4 (465.4)	2131.8 (424.4)	2252.2 (434.6)	2504.6 (496.7)	< 0.001
Protein, g/day	56.5 (17.1)	64.6 (15.7)	68.3 (16.0)	78.0 (19.2)	< 0.001
Fat, g/day	72.1 (29.8)	76.4 (28.4)	74.4 (28.3)	74.3 (31.1)	< 0.001
Carbohydrate, g/day	237.8 (80.1)	296.3 (75.9)	327.4 (78.8)	381.0 (102.5)	< 0.001
Sodium, g/day	4.5 (2.9)	5.0 (2.9)	5.2 (3.1)	5.4 (3.1)	< 0.001
Potassium, g/day	1.3 (0.4)	1.5 (0.3)	1.7 (0.4)	2.1 (0.8)	< 0.001
Sodium-to-potassium ratio	3.7 (2.5)	3.3 (2.0)	3.1 (1.9)	2.7 (1.6)	< 0.001

Variables are presented as mean (SD) or *n* (%)

### Relations of dietary total insoluble fiber and insoluble fibers from different foods with new-onset hypertension

During a median follow-up duration of 6.1 years (25th–75th: 3.7–11.4 years, 94,918 person-years in total), a total of 4252 (35.1%) participants developed new-onset hypertension.

Overall, there was an L-shaped relation of dietary total insoluble fiber with new-onset hypertension (Additional file 1: Figure S2). Accordingly, when dietary total insoluble fiber intake was assessed as quartiles, the adjusted HRs (95% CIs) of new-onset hypertension were 1.00 (reference), 0.78 (0.60, 1.01), 0.62 (0.47, 0.83), and 0.61 (0.44, 0.85) across the quartiles of total insoluble fiber intake, respectively. The risk of new hypertension seemed to reach a plateau when the dietary total insoluble fiber intake was greater than 9.6 g/day (quartile 3) (Table 2). Further adjustments for dietary intakes of vitamin A, vitamin B_2_, niacin, copper, and zinc (model 2); dietary intakes of refined and whole grains, vegetables, beans, tubers, and fruits (model 3); or BMI trajectory (model 4) did not substantially change the results (Additional file 1: Table S3). Similar results were also observed when dietary total insoluble fiber intake was estimated by MSM (Additional file 1: Figure S3) or exclusion of those with physician-diagnosed hypertension or receiving antihypertensive treatments (Additional file 1: Figure S4). Moreover, we further investigated the relation of changes in dietary total insoluble fiber intake in the first two waves with new-onset hypertension that occurred in subsequent follow-up waves. As expected, although not statistically significant, participants who consumed dietary total insoluble fiber ≥ 9.6 g/day in both waves had the lowest risk of new hypertension compared with those who consumed total insoluble fiber < 9.6 g/day in both diets (HR, 0.68; 95% CI: 0.43–1.06) (Additional file 1: Table S4).

**Table 2:** The association of total dietary insoluble fiber with new-onset hypertension

**Dietary insoluble fiber, g/day**	***N***	**Cases (rate)**^a^	**Crude model**		**Adjusted model**^b^	
			**HR (95%CI)**	***P* value**	**HR (95%CI)**	***P* value**
Quartile						
Q1 (< 7.1)	3033	973 (53.2)	Ref		Ref	
Q2 (7.1–9.6)	3032	984 (39.4)	0.72 (0.66, 0.79)	< 0.001	0.78 (0.60, 1.01)	0.062
Q3 (9.6–12.9)	3033	1040 (38.9)	0.71 (0.65, 0.78)	< 0.001	0.62 (0.47, 0.83)	0.001
Q4 (≥ 12.9)	3033	1255 (50.4)	0.93 (0.85, 1.01)	0.074	0.61 (0.44, 0.85)	0.003

^a^Incidence rate was presented as per 1000 person-years

^b^Adjusted for sex, body mass index, systolic blood pressure, diastolic blood pressure, smoking, alcohol drinking, urban/rural residents, regions, education levels, occupations, diabetes, physical activity levels, dietary intakes of sodium, potassium, protein, fat, and carbohydrate

**Fig. 1:**
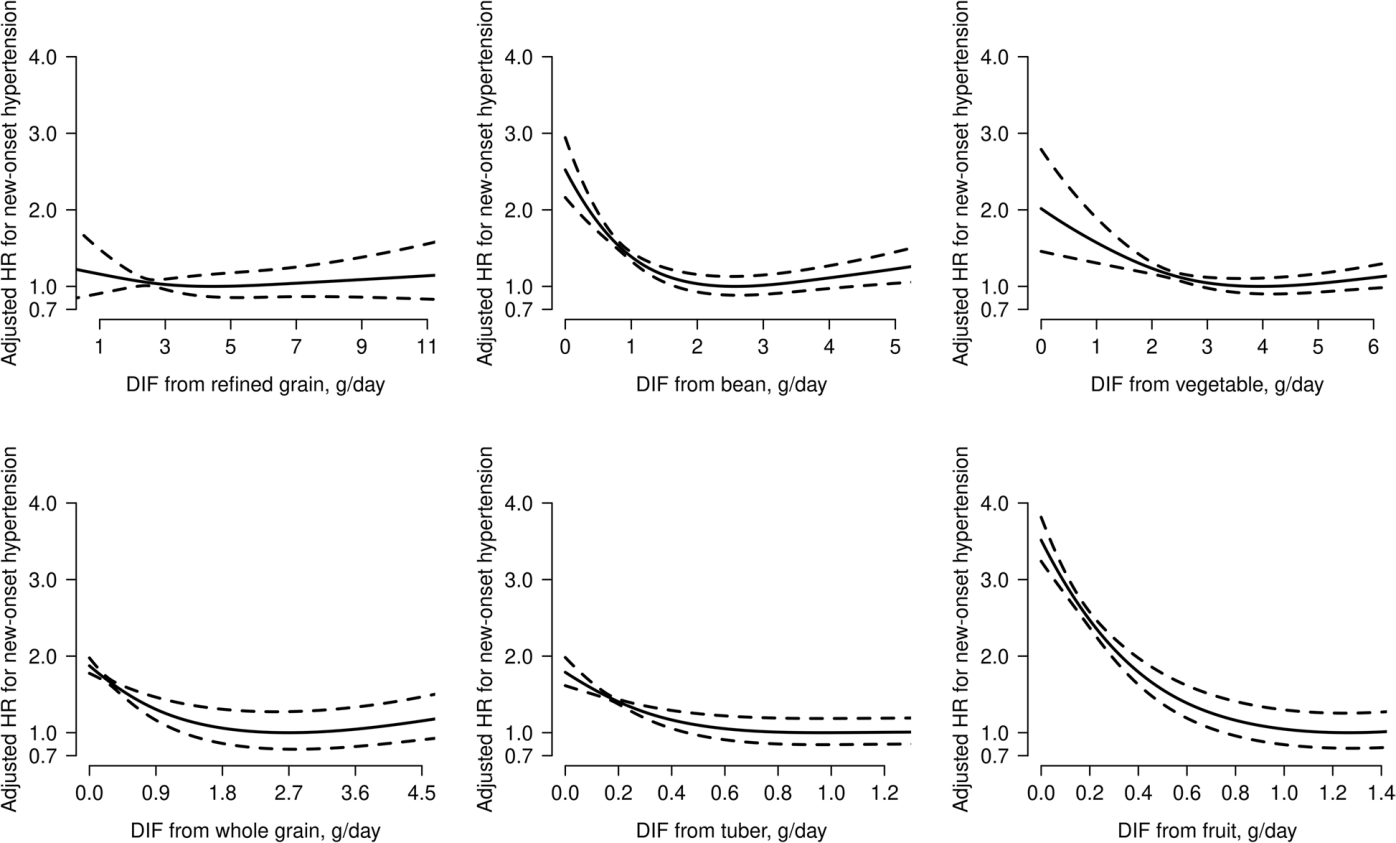
The associations between intakes of specific-sourced insoluble fiber with new-onset hypertension. Adjusted for sex, body mass index, systolic blood pressure, diastolic blood pressure, smoking, alcohol drinking, urban/rural residents, regions, education levels, occupations, diabetes, physical activity levels, dietary intakes of sodium, potassium, protein, fat, and carbohydrate, as well as mutual adjustments for intake from other specific dietary insoluble fiber source. DIF, dietary insoluble fiber

Figure 1 showed the associations between dietary insoluble fibers derived from different food sources and new-onset hypertension. There were L-shaped associations of insoluble fibers derived from vegetables, beans, tubers, and fruits with new-onset hypertension and a reversed J-shaped association of whole grain-derived insoluble fiber with new-onset hypertension (all *P* values for nonlinearity < 0.05). That is, for each above insoluble fiber, there is a window of consumption (appropriate level) where the risk of new-onset hypertension was lowest. However, there was no obvious association between refined grain-derived insoluble fiber and new-onset hypertension.

Similar trends were found when assessing different sources of insoluble fiber intakes as categorical variables. Accordingly, the appropriate levels (g/day) of specific sourced insoluble fiber associated with the lowest risk of new-onset hypertension were $\ge$ 1.9 (the 2–4 quartiles) for vegetable-derived insoluble fiber, $\ge$ 0.4 (the 2–4 quartiles) for bean-derived insoluble fiber, 0– < 1.7 (the 1–2 tertiles among consumers) for whole grain-derived insoluble fiber, > 0 (consumers) for tuber-derived insoluble fiber, and > 0 (consumers) for fruit-derived insoluble fiber (Additional file 1: Figure S5).

### Relation of variety score of insoluble fiber sources with new-onset hypertension

Since there was no obvious relation of refined grain-derived insoluble fiber with new-onset hypertension, the refined grain was not included in the calculation of variety score of insoluble fiber sources. As such, if a participant consumed one of the 5 major food sources (vegetables, beans, tubers, fruits, and whole grain) of insoluble fiber at an appropriate quantity during the entire study period, he/she will get one point, with a maximal score of 5.

**Fig. 2:**
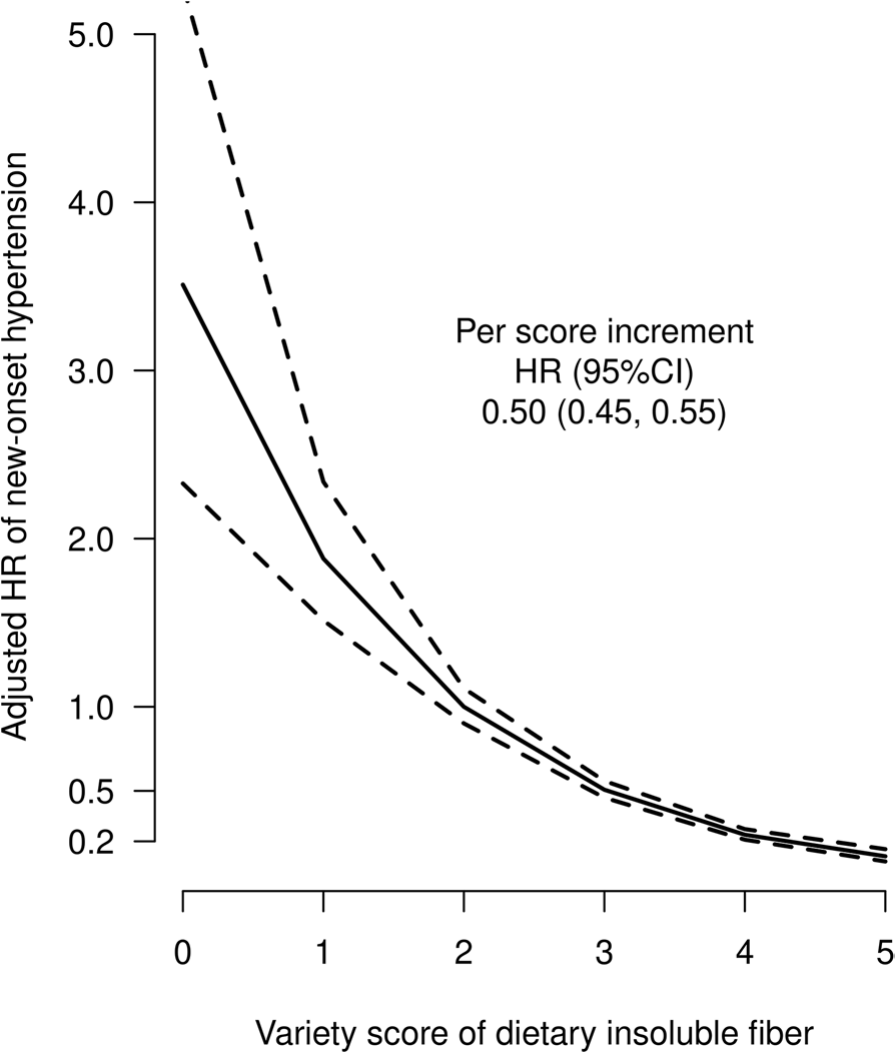
The association between variety score of dietary insoluble fiber sources and new-onset hypertension. Adjusted for sex, body mass index, systolic blood pressure, diastolic blood pressure, smoking, alcohol drinking, urban/rural residents, regions, education levels, occupations, diabetes, physical activity levels, dietary intakes of sodium, potassium, protein, fat, and carbohydrate, as well as total insoluble fiber

Overall, there was an inverse relation of the variety score of insoluble fiber sources with new-onset hypertension (per score increment, HR, 0.50; 95% CI, 0.45–0.55) (Fig. 2). Further adjustments for variety score of dietary protein sources (model 2); dietary intakes of vitamin A, vitamin B_2_, niacin, copper, and zinc (model 3); dietary intakes of refined and whole grains, vegetables, beans, tubers, and fruits (model 4); or BMI trajectory classes (model 5) did not substantially change the results (Additional file 1: Table S5). Similar trends were also observed after the removal of any one kind of insoluble fiber from the variety score of insoluble fiber (Additional file 1: Table S6). Moreover, among participants with blood sample measurements, further adjustments for creatinine concentrations also did not materially alter the results (Additional file 1: Figure S5).

In the stratified analyses, the inverse associations between variety score of insoluble fiber sources (per score increment) and the risk of new-onset hypertension were found in all the subgroups. Although the *P* value of the interaction for fat intake was < 0.05, because of multiple testing and similar directionality of the associations in different subgroups, the clinical implications of these interactions still should be further investigated (Additional file 1: Table S6).

## Discussion

In this large, prospective cohort study among general Chinese adults, we reported an L-shaped association between dietary total insoluble fiber and new-onset hypertension. In addition, there was no obvious association of refined grain-derived insoluble fiber with new-onset hypertension, while a reversed J-shaped association of whole grain-derived insoluble fiber and L-shaped associations for insoluble fibers from vegetables, beans, tubers, and fruits, with new-onset hypertension. Moreover, a higher variety score of insoluble fibers was significantly associated with a lower risk of new-onset hypertension.

A previous meta-analysis of 18 RCTs reported that an increase in total fiber intake had no significant impact on BP [9]. Another meta-analysis of 15 RCTs with the moderate quality found that higher intakes of total dietary fiber were associated with lower SBP levels (mean difference: − 1.27 mmHg; 95% CI: − 2.50 to − 0.04) [25]. However, among these RCTs, limited data were available for fiber described as soluble or insoluble. The potential effect and the underlying mechanisms of soluble fiber on BP have been relatively thoroughly investigated [9, 26]. Nevertheless, to date, only two cross-sectional studies have evaluated the relation of insoluble fiber intake and the prevalence of hypertension and reported inconsistent findings [10, 27]. As such, to date, the prospective relations of the variety and quantity of different sources of dietary insoluble fibers intake and hypertension remain uncertain. Our current study, with a prospective design and a relatively large sample size, provided an opportunity to assess the dose–response associations between the variety and quantity of insoluble fibers intake from different food sources and new-onset hypertension in the general population.

Our study provides some new insights. First, there were non-linear relations of dietary total insoluble fiber and dietary insoluble fibers from different food sources with new-onset hypertension, including a reversed J-shaped association for the whole grain-derived insoluble fiber, and L-shaped associations for the total insoluble fiber, and insoluble fibers from vegetables, beans, tubers, and fruits. That is, when these foods-derived insoluble fiber intakes were relatively low, there were negative correlations between intakes of foods-derived insoluble fiber and hypertension risk; however, when intake exceeded certain thresholds, the risks of new-onset hypertension will increase or reach a plateau. More importantly, we found that there was no obvious association between refined grain-derived insoluble fiber and hypertension. The potential mechanisms by which insoluble fiber lowers blood pressure may include reduced inflammation levels [28, 29] and improved endothelial function [30]. Moreover, cellulose could inhibit starch digestion by binding α- amylase [31], thereby reducing glucose absorption and improving insulin resistance, and lowering the risk of hypertension [32]. However, the reversed J-shaped association of whole grain-derived insoluble fiber with new-onset hypertension and the no obvious association of refined grain-derived insoluble fiber with new-onset hypertension indicated that too high intakes of grain-derived insoluble fibers, especially refined grain-derived insoluble fiber, may have no benefit on BP. Consistently, the smooth curve of the association between grain fiber intake and SBP levels also showed a U-shape in the Study of Women’s Health Across the Nation (SWAN) [33]. And the Tehran Lipid and Glucose Study found that there were positive relations of grain fiber with CVD risk score at baseline and increased insulin resistance index during the study follow-up [34]. It has been reported that the fiber of refined grain derived from cell walls in the starchy endosperm is nutrient-poor, as it does not include the biochemicals found in the nutrient-rich bran and germ [35]. Moreover, a recent study showed plasma trimethylamine N-oxide (TMAO) directly correlated with the intake of whole-grain products [36]. Overall, more studies are needed to confirm our findings and further examine the underlying mechanisms involved in the associations between the intake of different insoluble fibers and hypertension.

Second, we first found that there was a significant inverse association between the variety score of insoluble fiber sources and new-onset hypertension. More importantly, the removal of any one kind of fiber from the insoluble variety scores could not substantially change our findings. The possible explanation may be that the role of dietary fiber not only depends on the amount of intake but also its food sources. Since not all fibers from different food sources behave in the same way, fibers from a diverse range of sources, with differences in fiber structure and activities, may offer diverse functional characteristics and thus provide better health benefits. However, the detailed mechanisms still need to be further investigated in future studies.

There are some limitations needed to be mentioned. First, although a broad array of covariates had been adjusted in the regression models, the possibility of residual confounding cannot be excluded. For example, CHNS only collected information about self-reported physician-diagnosed hypertension, diabetes, asthma, and CVD but not kidney diseases at baseline. Therefore, we could not examine whether baseline kidney diseases may affect our findings. However, among those with blood sample measurements, further adjustments for creatinine concentrations did not substantially change our findings. Second, the neutral detergent fiber method is a reliable analytical tool for measuring insoluble fiber. We could not calculate the amount of soluble fiber intake. Third, our study only included insoluble fibers from 6 major food sources in Chinese adults. Fourth, since the detailed sampling information was not provided by CHNS, summary statistics like prevalence and incidence should be treated with caution. Finally, our study was conducted in the Chinese population, whether the findings can be extrapolated to other populations needs further investigation. Therefore, the results need further confirmation.

## Conclusions

In summary, our study suggested that there was an inverse relation of the variety of insoluble fibers with appropriate quantity from different food sources (whole grain, vegetable, bean, tuber, and fruit) and new-onset hypertension in general Chinese adults. If our results are further confirmed, these findings support that the intake of an appropriate quantity of insoluble fibers from various food sources plays an important role in the primary prevention of hypertension.

## Supplementary Information

**Additional file 1:** **Figure S1.** Flow chart of study participants. **Figure S2.** The relationship of total dietary insoluble fiber intake with new-onset hypertension^*^. **Figure S3.** The relationship of total dietary insoluble fiber intake estimated by multiple source method with new-onset hypertension^*^. **Figure S4.** The relationship of total dietary insoluble fiber intake with new-onset hypertension after excluding those with physician-diagnosed hypertension or receiving antihypertensive treatment. **Figure S5.** Forest plots of the relationship of specific-sourced dietary insoluble fiber intake with new-onset hypertension. **Figure S6.** The association between variety score of dietary insoluble fiber sources and new-onset hypertension further adjusted for serum creatinine*. **Table S1.** Characteristics of the participants with and without blood pressure data. **Table S2.** Food sources of dietary insoluble fiber intake. **Table S3.** Sensitivity analysis for the association between total dietary insoluble fiber intake (g/d) and new-onset hypertension. **Table S4.** The association between total dietary insoluble fiber intake (g/d) in different waves and new-onset hypertension. **Table S5.** Sensitivity analysis for the association between variety score of insoluble fiber source and new-onset hypertension. **Table S6.** The association between variety score of insoluble fiber source and new-onset hypertension after the removal of any one kind of insoluble fiber from the insoluble fiber variety score. **Table S7.** Stratified analyses of the association between the variety score of insoluble fiber source and new-onset hypertension.

## Data Availability

The datasets generated and analyzed during the current study are available the CHNS official website (http://www.cpc.unc.edu/projects/china).

## Abbreviations

BP: Blood pressure
BMI: Body mass index
CVD: Cardiovascular disease
CHNS: China health and nutrition survey
DBP: Diastolic blood pressure
FCTs: Food composition tables
MSM: Multiple source methods
RCTs: Randomized control trials
SD: Standardized deviation
SBP: Systolic blood pressure
TMAO: Trimethylamine n-oxide
