# Supplementary material for: Variety and quantity of dietary insoluble fiber intake from different sources and risk of new-onset hypertension
Source: BMC Med. 2023 Feb 16;21:61. doi: 10.1186/s12916-023-02752-7 (PMC9933403; doi:10.1186/s12916-023-02752-7)
Supplement: Supplementary file 1 — Additional file 1: Figure S1. Flow chart of study participants. Figure S2. The relationship of total dietary insoluble fiber intake with new-onset hypertension*. Figure S3. The relationship of total dietary insoluble fiber intake estimated by multiple source method with new-onset hypertension*. Figure S4. The relationship of total dietary insoluble fiber intake with new-onset hypertension after excluding those with physician-diagnosed hypertension or receiving antihypertensive treatment. Figure S5. Forest plots of the relationship of specific-sourced dietary insoluble fiber intake with new-onset hypertension. Figure S6. The association between variety score of dietary insoluble fiber sources and new-onset hypertension further adjusted for serum creatinine*. Table S1. Characteristics of the participants with and without blood pressure data. Table S2. Food sources of dietary insoluble fiber intake. Table S3. Sensitivity analysis for the association between total dietary insoluble fiber intake (g/d) and new-onset hypertension. Table S4. The association between total dietary insoluble fiber intake (g/d) in different waves and new-onset hypertension. Table S5. Sensitivity analysis for the association between variety score of insoluble fiber source and new-onset hypertension. Table S6. The association between variety score of insoluble fiber source and new-onset hypertension after the removal of any one kind of insoluble fiber from the insoluble fiber variety score. Table S7. Stratified analyses of the association between the variety score of insoluble fiber source and new-onset hypertension. [file 12916_2023_2752_MOESM1_ESM.docx]

**Additional File 1**

**Figure S1.** Flow chart of study participants

**Figure S2.** The relationship of total dietary insoluble fiber intake with new-onset hypertension^*^

**Figure S3.** The relationship of total dietary insoluble fiber intake estimated by multiple source method with new-onset hypertension^*^

**Figure S4.** The relationship of total dietary insoluble fiber intake with new-onset hypertension after excluding those with physician-diagnosed hypertension or receiving antihypertensive treatment

**Figure S5.** Forest plots of the relationship of specific-sourced dietary insoluble fiber intake with new-onset hypertension

**Figure S6.** The association between variety score of dietary insoluble fiber sources and new-onset hypertension further adjusted for serum creatinine *

**Table S1.** Characteristics of the participants with and without blood pressure data

**Table S2.** Food sources of dietary insoluble fiber intake

**Table S3.** The association between total dietary insoluble fiber intake (g/d) in different waves and new-onset hypertension

**Table S4.** Sensitivity analysis for the association between total dietary insoluble fiber intake (g/d) and new-onset hypertension

**Table S5.** Sensitivity analysis for the association between variety score of insoluble fiber source and new-onset hypertension

**Table S6.** The association between variety score of insoluble fiber source and new-onset hypertension after the removal of any one kind of insoluble fiber from the insoluble fiber variety score

**Table S7.** Stratified analyses of the association between the variety score of insoluble fiber source and new-onset hypertension


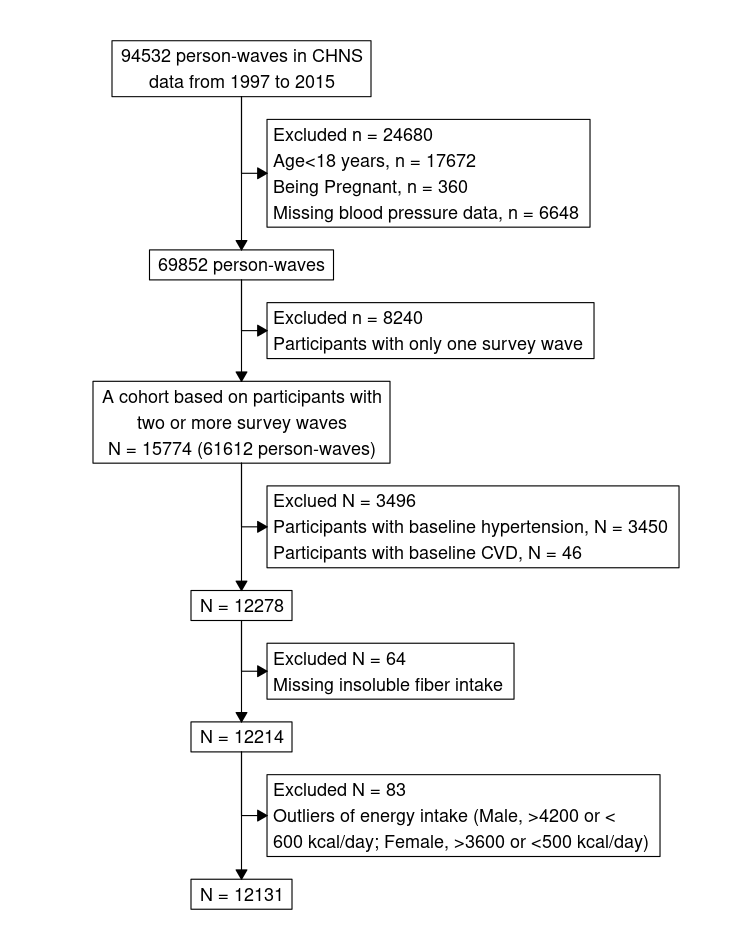


**Figure S1. Flow chart of study participants**


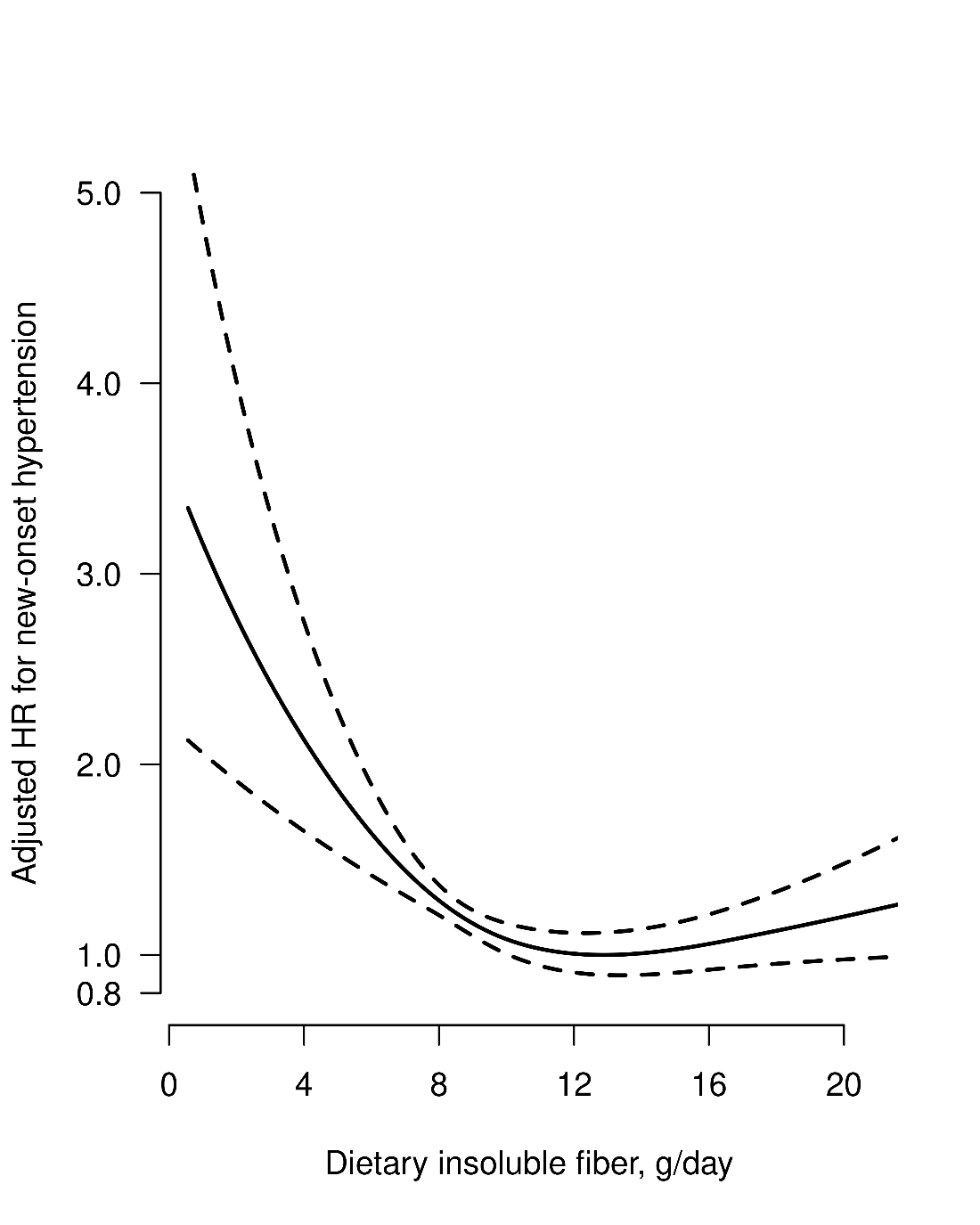


**Figure S2. The relationship of total dietary insoluble fiber intake with new-onset hypertension^*^**

^*^ Adjusted for sex, body mass index, systolic blood pressure, diastolic blood pressure, smoking, alcohol drinking, urban/rural, region, education, occupation, diabetes, physical activity, dietary intakes of sodium, potassium, protein, fat, and carbohydrate.


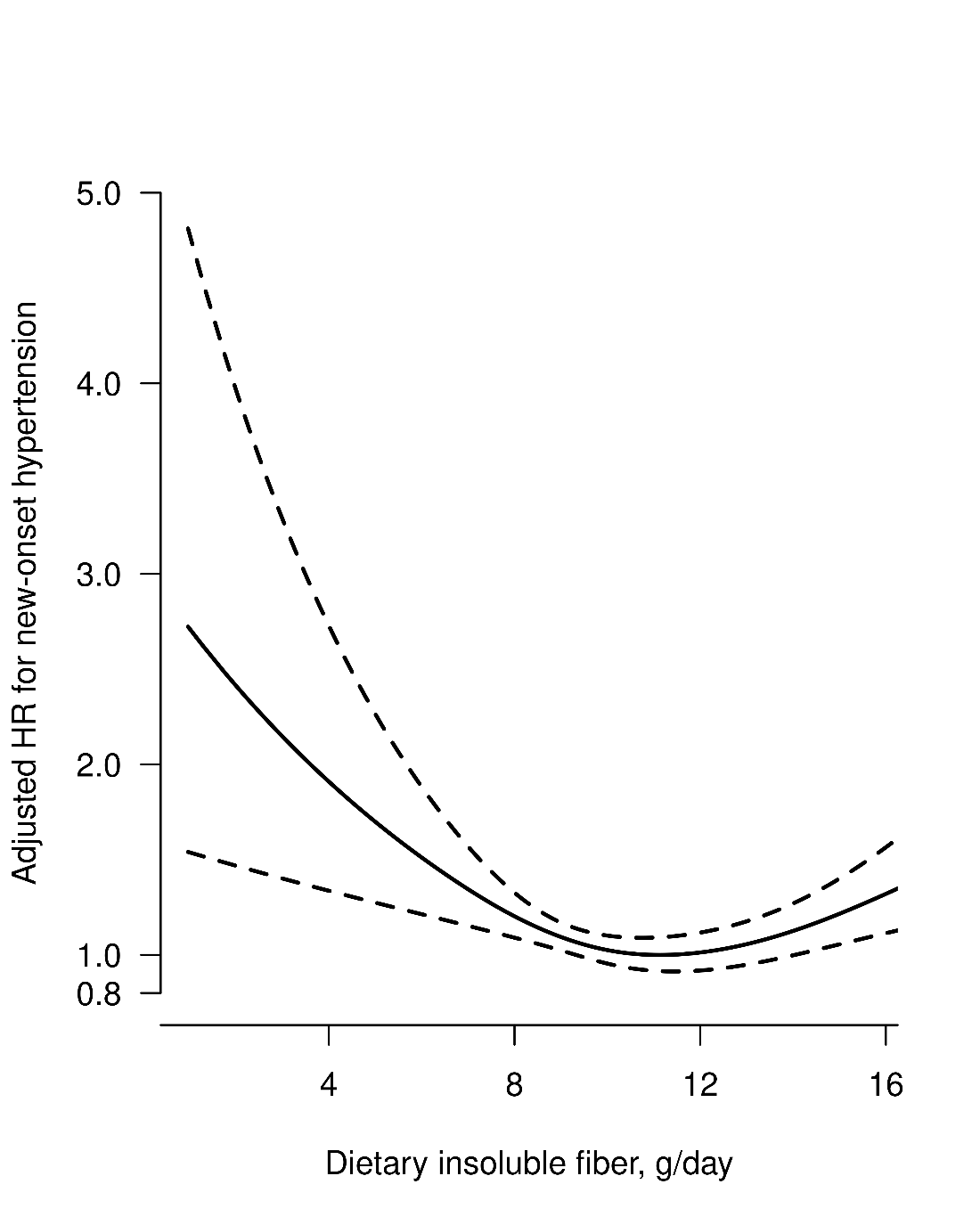


**Figure S3. The relationship of total dietary insoluble fiber intake estimated by multiple source method with new-onset hypertension^*^**

^*^ Adjusted for sex, body mass index, systolic blood pressure, diastolic blood pressure, smoking, alcohol drinking, urban/rural, region, education, occupation, diabetes, physical activity, dietary intakes of sodium, potassium, protein, fat, and carbohydrate.


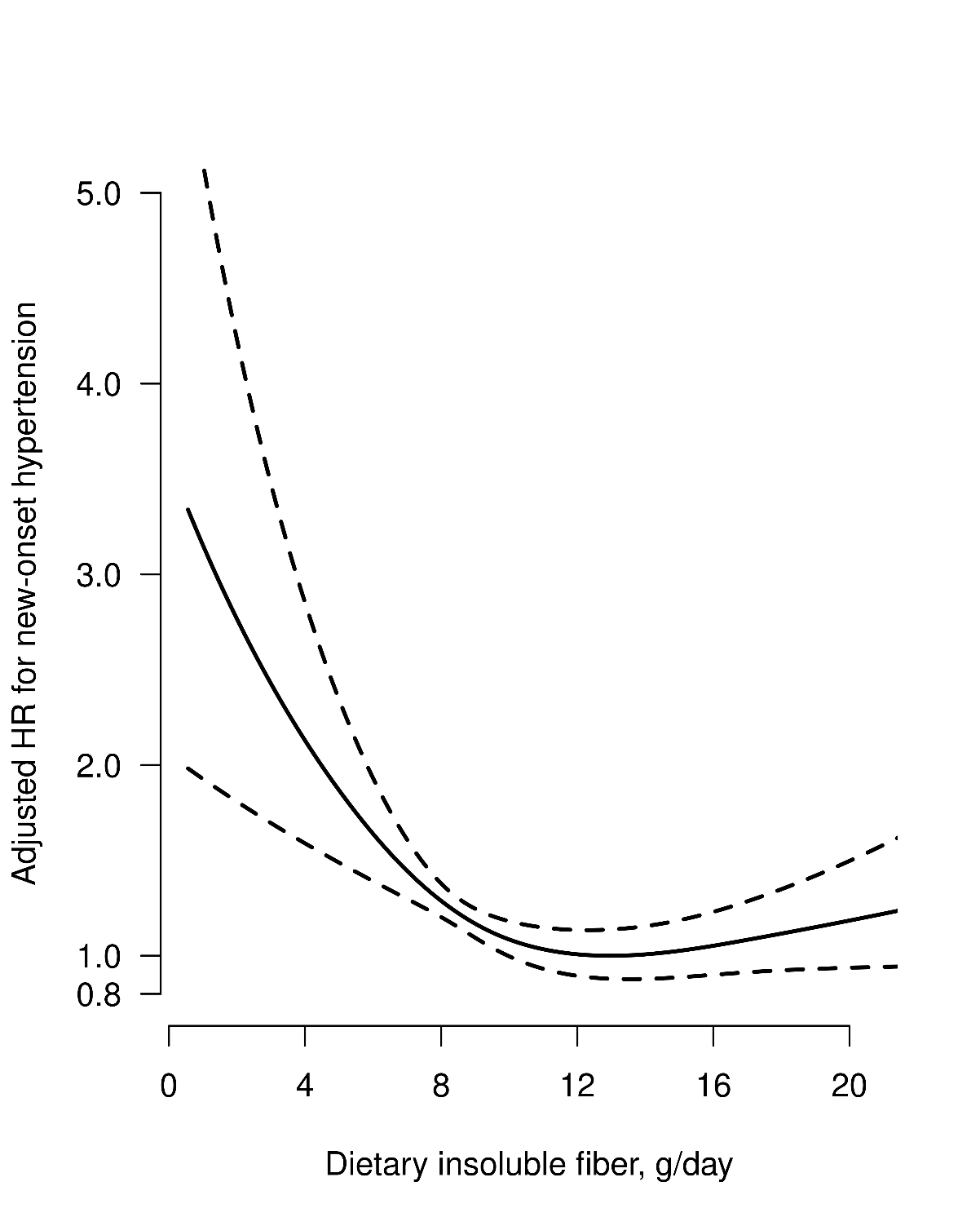
**Figure S4. The relationship of total dietary insoluble fiber intake with new-onset hypertension after excluding those with physician-diagnosed hypertension or receiving antihypertensive treatment^*^**

^*^ Adjusted for sex, body mass index, systolic blood pressure, diastolic blood pressure, smoking, alcohol drinking, urban/rural, region, education, occupation, diabetes, physical activity, dietary intakes of sodium, potassium, protein, fat, and carbohydrate.


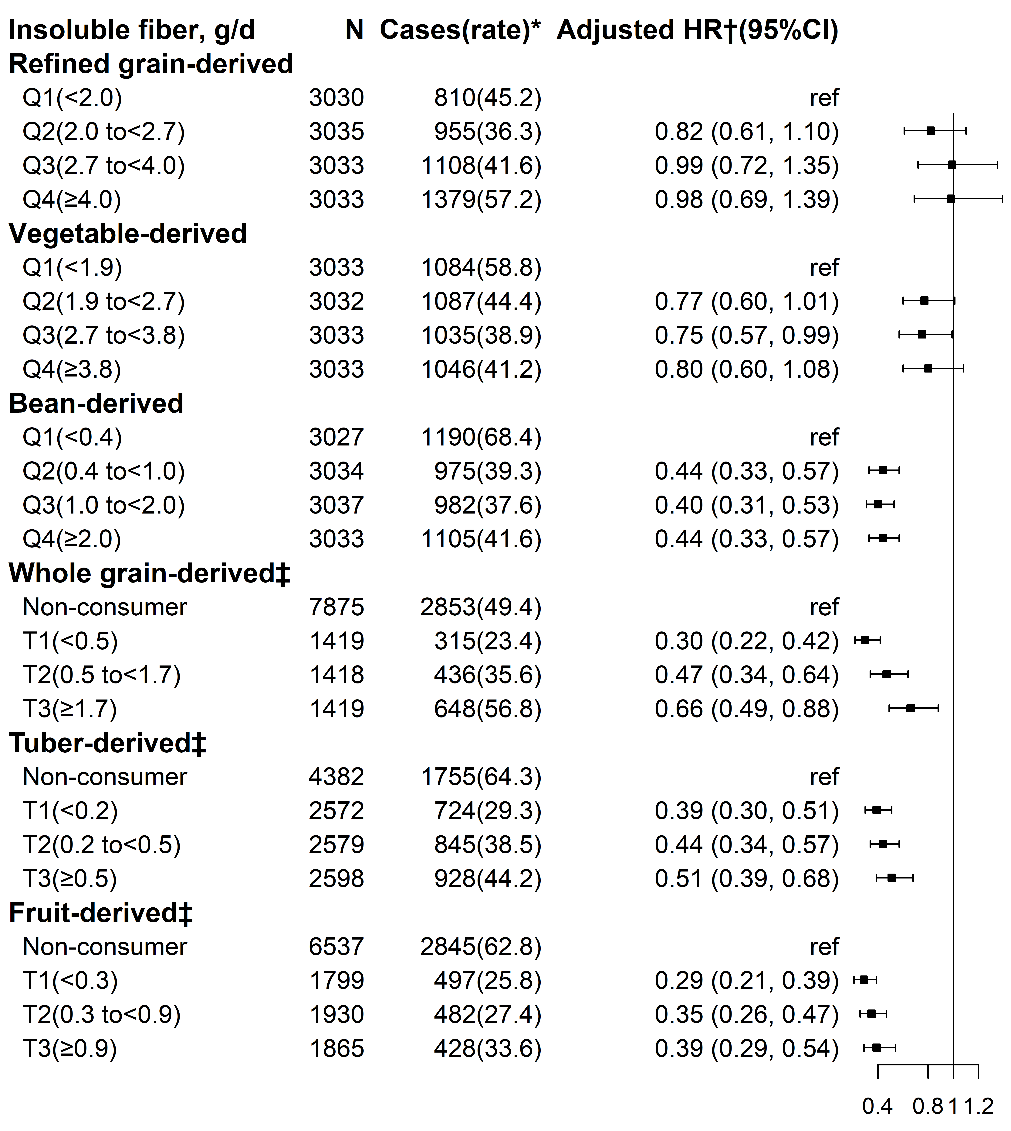


**Figure S5. Forest plots of the relationship of specific-sourced dietary insoluble fiber intake with new-onset hypertension**

*Incidence rates was presented as per 1000 person-years

†Adjusted model: adjusted for sex, body mass index, systolic blood pressure, diastolic blood pressure, smoking, alcohol drinking, urban/rural, region, education, occupation, diabetes, physical activity, dietary intakes of sodium, potassium, protein, fat, and carbohydrate and mutual adjustments for intake from other specific dietary insoluble fiber source.

‡For these dietary insoluble fibers whose proportion of non-consumers was over 20%, consumers were divided into three groups according to tertiles and non-consumers were used as the reference.


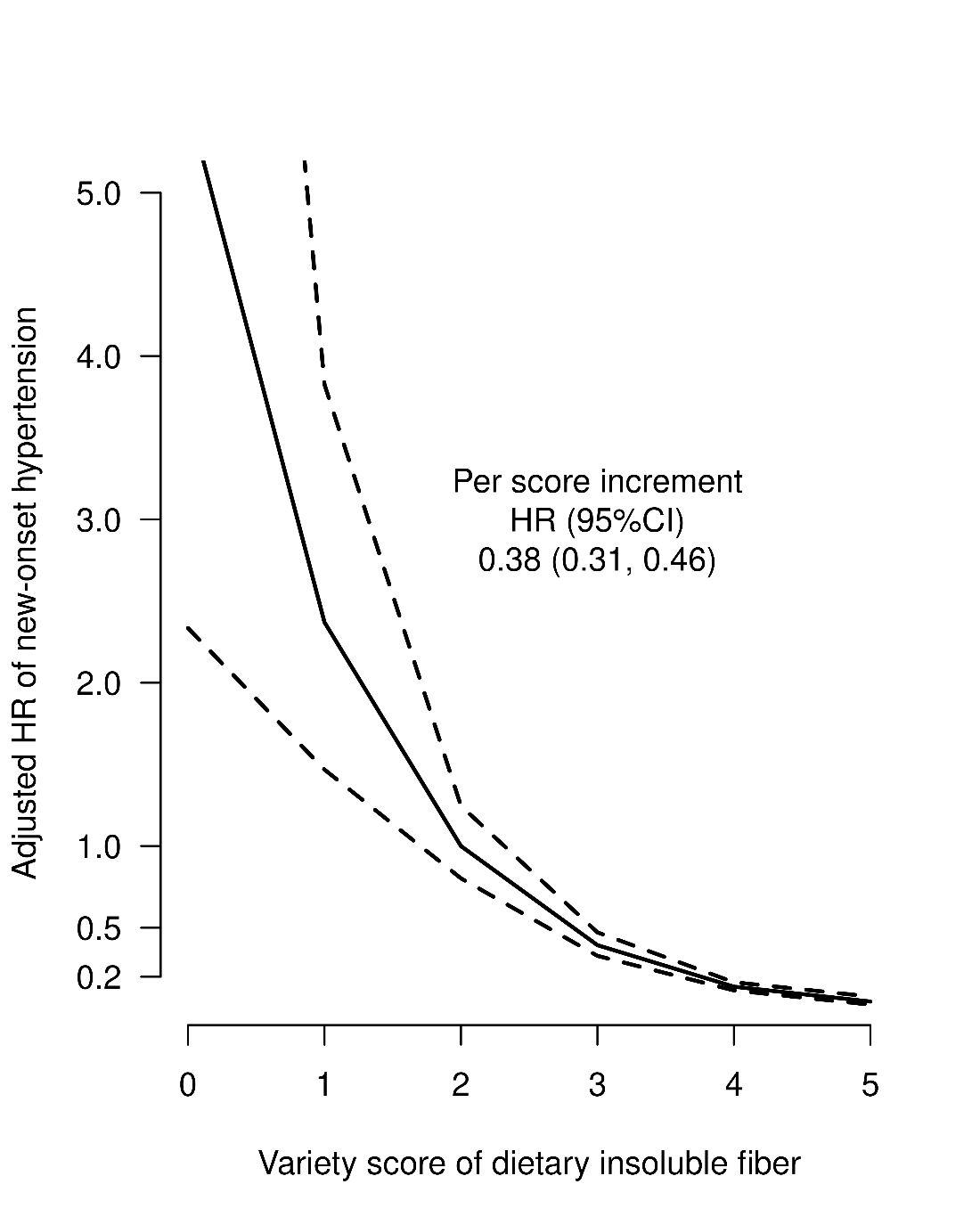


**Figure S6. The association between variety score of dietary insoluble fiber sources and new-onset hypertension ***

*Adjusted for sex, body mass index, systolic blood pressure, diastolic blood pressure, smoking, alcohol drinking, urban/rural residents, regions, education levels, occupations, diabetes, physical activity levels, dietary intakes of sodium, potassium, protein, fat, and carbohydrate, total insoluble fiber, serum creatinine

**Table S1.** **Characteristics of the participants with and without blood pressure data**

| Characteristics | Those with BP data | Those without BP data |
| --- | --- | --- |
| N, person-wave | 69852 | 6648 |
| Age, y | 49.3 (15.6) | 41.5 (17.5) |
| Man, n (%) | 33048 (47.3) | 3800 (57.2) |
| Body mass index, kg/m2 | 23.3 (3.5) | 24.1 (5.5) |
| Smoking, n (%) | 21314 (30.6) | 1905 (34.0) |
| Alcohol drinking, n (%) | 22705 (32.7) | 1944 (35.2) |
| Urban resident, n (%) | 24324 (34.8) | 2246 (33.8) |
| Regions, n (%) |  |  |
| Central | 32416 (46.4) | 3726 (56.0) |
| North | 14774 (21.2) | 831 (12.5) |
| South | 22662 (32.4) | 2091 (31.5) |
| Occupation, No. (%) |  |  |
| Farmer | 19120 (27.7) | 1304 (19.9) |
| Worker | 6585 (9.5) | 1217 (18.6) |
| Unemployed | 27249 (39.4) | 2049 (31.3) |
| Other | 16174 (23.4) | 1978 (30.2) |
| Education, n (%) |  |  |
| Illiteracy | 13515 (20.1) | 771 (12.2) |
| Primary school | 13434 (20.0) | 1005 (15.9) |
| Middle school | 21570 (32.1) | 2280 (36.2) |
| High school or above | 18753 (27.9) | 2248 (35.7) |
| Physical activity, n (%) |  |  |
| Low | 16695 (24.1) | 1623 (29.4) |
| Moderate | 25697 (37.0) | 1754 (31.8) |
| Vigorous | 27000 (38.9) | 2146 (38.9) |

Variables are presented as Mean (SD) or person-wave (%).

**Table S2. Food sources of dietary insoluble fiber intake**

| Categories  (percent of total insoluble fiber） | Items |
| --- | --- |
| Refined grain (32%) | Refined wheat flour, grain noodle, wheat bun, wheat bread, wheat pancake, wheat gluten, and rice, etc. |
| Vegetable (30%) | Cruciferous vegetables, Leguminous vegetables, Cucurbitaceous and solanaceous vegetables, Green leafy vegetables, Fungi and algae, etc. Excluding canned products. |
| Bean (14%) | Soybean, soybean flour, soybean milk, soybean curd, soy harm, soy chicken, soy intestine, soy shrimp, mung bean, adzuki bean, kidney bean, broad bean, hyacinth bean, cowpea, garden pea, gorse, chickpeas. Excluding fermented soybean products, etc. |
| Whole grain (6%) | Wheat grain, wheat bran, corn, barley, millet, sorghum, buckwheat, oat, and adlay, etc. |
| Tuber (5%) | Potato, potato flour, cassava, broad bean starch, pea starch, corn starch, lotus root starch, and starch noodle, etc. |
| Fruit (4%) | Kernel fruit, drupe fruit, berry, orange fruit, tropic fruit, and melon, etc. Excluding canned products or juice. |

**Table S3. Sensitivity analysis for the association between total dietary insoluble fiber intake (g/d) and new-onset hypertension**

| **Dietary total insoluble**  **fiber (g/day)** | **Model 1** |  | **Model 2** |  | **Model 3** |  | **Model 4** |  |
| --- | --- | --- | --- | --- | --- | --- | --- | --- |
|  | **HR (95%CI)** | ***P* value** | **HR (95%CI)** | **P value** | **HR (95%CI)** | ***P* value** | **HR (95%CI)** | ***P* value** |
| Quartiles |  |  |  |  |  |  |  |  |
| Q1(< 7.1) | ref |  | ref |  | ref |  | ref |  |
| Q2(7.1 - 9.6) | 0.78 (0.60, 1.01) | 0.062 | 0.76 (0.58, 0.99) | 0.044 | 0.82 (0.63, 1.08) | 0.156 | 0.78 (0.60, 1.01) | 0.06 |
| Q3(9.6 - 12.9) | 0.62 (0.47, 0.83) | 0.001 | 0.61 (0.45, 0.82) | 0.001 | 0.72 (0.53, 0.97) | 0.031 | 0.62 (0.47, 0.83) | 0.001 |
| Q4(≥12.9) | 0.61 (0.44, 0.85) | 0.003 | 0.62 (0.44, 0.87) | 0.006 | 0.74 (0.52, 1.05) | 0.094 | 0.61 (0.44, 0.84) | 0.002 |

Model 1: Adjusted for sex, body mass index, systolic blood pressure, diastolic blood pressure, smoking, alcohol drinking, urban/rural, region, education, occupation, diabetes , physical activity, dietary intakes of sodium, potassium, protein, fat, and carbohydrate.

Model 2: Adjusted for variables in model 1, as well as dietary intakes of vitamin A, vitamin B2, niacin, copper, zinc.

Model 3: Adjusted for variables in model 1, as well as dietary intakes of refined and whole grains, vegetables, beans, tubers and fruits.

Model 4: Adjusted for variables in model 1, as well as body mass index trajectory

**Table S4. The association between total dietary insoluble fiber intake (g/d) in different waves and new-onset hypertension**

| **Dietary total insoluble fiber (g/day)** | | **Model 1^*^** |  | **Model 2^*^** |  |
| --- | --- | --- | --- | --- | --- |
|  |  | **HR (95%CI)** | ***P* value** | **HR (95%CI)** | **P value** |
| Wave 1 | Wave 2 |  |  |  |  |
| <9.6 | <9.6 | ref |  | ref |  |
| <9.6 | ≥9.6 | 0.82 (0.54, 1.25) | 0.358 | 0.82 (0.54, 1.24) | 0.349 |
| ≥9.6 | <9.6 | 0.89 (0.61, 1.29) | 0.532 | 0.77 (0.50, 1.19) | 0.236 |
| ≥9.6 | ≥9.6 | 0.78 (0.52, 1.15) | 0.209 | 0.68 (0.43, 1.06) | 0.085 |

^*^Model 1: Adjusted for sex, body mass index, systolic blood pressure, diastolic blood pressure, smoking, alcohol drinking, urban/rural residents, regions, education levels, occupations, diabetes , physical activity levels, dietary intakes of sodium, potassium, protein, fat, and carbohydrate

Model 2: adjusted for all variables in model 1 plus the total dietary insoluble fiber in wave 1.

**Table S5. Sensitivity analysis for the association between variety score of insoluble fiber source and new-onset hypertension**

| **Variety score of insoluble fiber** | **HR (95%CI)** | ***P* value** |
| --- | --- | --- |
| **(Per score increment)** |  |  |
| **Model 1** | 0.50 (0.45, 0.55) | <0.001 |
| **Model 2** | 0.55 (0.49, 0.61) | <0.001 |
| **Model 3** | 0.50 (0.45, 0.56) | <0.001 |
| **Model 4** | 0.48 (0.43, 0.54) | <0.001 |
| **Model 5** | 0.50 (0.45, 0.55) | <0.001 |

Model 1: Adjusted for sex, body mass index, systolic blood pressure, diastolic blood pressure, smoking, alcohol drinking, urban/rural, region, education, occupation, diabetes , physical activity, dietary intakes of sodium, potassium, protein, fat, and carbohydrate, as well as total insoluble fiber intake.

Model 2: Adjusted for variables in model 1, as well as variety score of dietary protein sources

Model 3: Adjusted for variables in model 1, as well as dietary intakes of vitamin A, vitamin B2, niacin, copper and zinc.

Model 4: Adjusted for variables in model 1, as well as dietary intakes of refined and whole grains, vegetables, beans, tubers and fruits

Model 5: Adjusted for variables in model 1, as well as body mass index trajectory

**Table S6. The association between variety score of insoluble fiber source and new-onset hypertension after the removal of any one kind of insoluble fiber from the insoluble fiber variety score**

| **Variety score of insoluble fiber** | **Crude model** |  | **Adjusted model^*^** |  |
| --- | --- | --- | --- | --- |
|  | HR (95%CI) | P value | HR (95%CI) | P value |
| **Excluding vegetable-derived insoluble fiber** | 0.61 (0.59, 0.63) | <0.001 | 0.46 (0.41, 0.51) | <0.001 |
| **Excluding bean-derived insoluble fiber** | 0.58 (0.56, 0.60) | <0.001 | 0.49 (0.43, 0.55) | <0.001 |
| **Excluding whole grain-derived insoluble fiber** | 0.60 (0.59, 0.62) | <0.001 | 0.49 (0.44, 0.55) | <0.001 |
| **Excluding tuber-derived insoluble fiber** | 0.59 (0.57, 0.61) | <0.001 | 0.50 (0.44, 0.56) | <0.001 |
| **Excluding fruit-derived insoluble fiber** | 0.64 (0.62, 0.66) | <0.001 | 0.53 (0.47, 0.59) | <0.001 |

**^*^**Adjusted for sex, body mass index, systolic blood pressure, diastolic blood pressure, smoking, alcohol drinking, urban/rural residents, regions, education levels, occupations, diabetes , physical activity levels, dietary intakes of sodium, potassium, protein, fat, and carbohydrate, as well as total insoluble fiber intake.

**Table S7. Stratified analyses of the association between the variety score of insoluble fiber source (per score increment) and new-onset hypertension**

| **Subgroups** | **N** | **Cases(rate^*^)** | **Adjusted HR**^†^**(95%CI)** | ***P* for interaction** |
| --- | --- | --- | --- | --- |
| **Age, yrs** |  |  |  | 0.256 |
| <60 | 10784 | 3480(39.5) | 0.51 (0.45, 0.56) |  |
| ≥60 | 1347 | 772(114.8) | 0.40 (0.27, 0.60) |  |
| **Sex** |  |  |  | 0.709 |
| Female | 6459 | 2076(40.1) | 0.49 (0.43, 0.56) |  |
| Male | 5672 | 2176(50.5) | 0.51 (0.44, 0.58) |  |
| **BMI, kg/m^2^** |  |  |  | 0.138 |
| <24 | 8772 | 2742(37.3) | 0.47 (0.42, 0.54) |  |
| ≥24 | 3276 | 1482(71.0) | 0.54 (0.47, 0.64) |  |
| **BP stages**‡ |  |  |  | 0.436 |
| Optimal | 1766 | 952(93.4) | 0.53 (0.43, 0.65) |  |
| Normal | 4087 | 1655(56.2) | 0.52 (0.45, 0.60) |  |
| High normal | 6278 | 1645(29.8) | 0.47 (0.40, 0.54) |  |
| **Energy, Kcal** |  |  |  | 0.407 |
| <2159 | 6065 | 1959(45.5) | 0.48 (0.42, 0.55) |  |
| ≥2159 | 6066 | 2293(44.2) | 0.52 (0.45, 0.59) |  |
| **Total fat intake, g/day** |  |  |  | 0.007 |
| <71 | 6065 | 2308(49.3) | 0.44 (0.38, 0.51) |  |
| ≥71 | 6066 | 1944(40.4) | 0.56 (0.49, 0.64) |  |
| **Total protein intake, g/day** |  |  |  | 0.625 |
| <65 | 6065 | 2103(45.7) | 0.49 (0.43, 0.56) |  |
| ≥65 | 6066 | 2149(43.9) | 0.51 (0.45, 0.59) |  |
| **Total carbohydrate intake, g/day** |  |  |  | 0.072 |
| <305 | 6065 | 1812(43.0) | 0.54 (0.47, 0.61) |  |
| ≥305 | 6066 | 2440(46.2) | 0.45 (0.39, 0.53) |  |
| **Total insoluble fiber intake, g/d** |  |  |  | 0.196 |
| <9.6 | 6065 | 1957(45.2) | 0.53 (0.46, 0.61) |  |
| ≥9.6 | 6066 | 2295(44.4) | 0.47 (0.41, 0.54) |  |
| **Sodium intake, g/d** |  |  |  | 0.436 |
| <4.4 | 6065 | 2055(45.8) | 0.48 (0.42, 0.56) |  |
| ≥4.4 | 6066 | 2197(43.9) | 0.52 (0.45, 0.59) |  |
| **Potassium intake, g/d** |  |  |  | 0.084 |
| <1.6 | 6065 | 2139(47.4) | 0.54 (0.47, 0.62) |  |
| ≥1.6 | 6066 | 2113(42.4) | 0.46 (0.40, 0.53) |  |

*Incidence rate was presented as per 1000 person-years

†Adjusted for sex, body mass index, systolic blood pressure, diastolic blood pressure, smoking, alcohol drinking, urban/rural residents, regions, education levels, occupations, diabetes , physical activity levels, dietary intakes of sodium, potassium, protein, and carbohydrate, as well as total insoluble fiber intake.

‡ Blood pressure (BP) stages: optimal: SBP <120 & DBP <80, normal: 120≤ SBP <130 & 80≤ DBP< 85, high normal: 130≤ SBP <140 & 85≤ DBP< 90 mmHg.
